# Evaluation of Zinc Plasma Level in Iranian Cirrhotic Patients due to Hepatitis B and Hepatitis C

**Published:** 2010-03-01

**Authors:** Mohammad Abbasi Nazari, Sahar Hasani Malayeri, Mohammad Amin Pourhoseingholi, Seyed Reza Mohebi, Mohammad Reza Zali

**Affiliations:** 1 Department of Clinical Pharmacy, School of Pharmacy, Shahid Beheshti University of Medical Sciences, Tehran, Iran; 2 Research Center for Gastrointestinal and Liver Diseases, Shahid Beheshti University of Medical Sciences, Tehran, Iran

**Keywords:** Zinc, Cirrhosis, Hepatitis B, Hepatitis C, Iran

## Abstract

**Background and Aims:**

Zinc (Zn) has various significant roles in physiological functions of the liver. Furthermore, it has been reported that the administration of zinc has an important role in pharmacotherapy of viral hepatitis. Cirrhotic patients with decrease in plasma zinc level have been covered in previous studies. It is seemingly necessary to assess the zinc level, in Iranian cirrhotic patients, as a distinct population, Because of the large phytate amounts in Iranians diet. Regarding to etiology, disease progress, and treatment, there are some differences in the 2 most common causes of cirrhosis in the Iranian population (hepatitis B and hepatitis C) and it is possible that the zinc level may be different between the two. This study was done to shadow some lights on the subject.

**Methods:**

Between April 2008 and November 2008, plasma zinc level was determined, by atomic absorption method, in 60 cirrhotic inpatients treated due to hepatitis B or hepatitis C in Talighani hospital (a referral center for gastrontestinal and liver diseases in Tehran, Iran).

**Results:**

Mean ± standard deviation (SD) plasma zinc levels determined 0.34±0.22 mg/L and 0.37±0.22 mg/L in hepatitis B and hepatitis C patients respectively. Analysis of t-test showed there is no significant difference between 2 groups regarding to plasma zinc level (P = 0.745).

**Conclusions:**

It is concluded that zinc level of studied cirrhotic patients is less than half of the normal range. Moreover, there is no difference in plasma zinc level between cirrhotic patients due to hepatitis B or hepatitis C. Regarding to this result, supplementation with complementary zinc, may be recommended in both groups in order to optimize the nutritional support and probably better the treatment response.

## Introduction

Among trace elements, zinc (Zn) is a micronutrient influencing growth and affecting the development and integrity of the immune system [1]. Also, it has important roles in physiological functions of the human body. It has a critical role for the function of over 300 enzymes [2]. Furthermore, it plays an important role in function of the liver. There are hepatic and extrahepatic actions for Zn in the prevention of alcoholic liver injury [3].

Zinc deficiency has been involved in the pathogenesis of a number of clinical findings in chronic liver disease. These include the possible role of Zn deficiency in the pathogenesis of hepatic encephalopathy, by inducing alterations in urea metabolism [4]. In a study, Gur et al. reported decreased level of plasma zinc in cirrhotic patients due to hepatitis B [5]. Hepatitis B and hepatitis C are the most common causes of liver cirrhosis in many countries like Iran [6]. As nutritional habits are different among various populations, it is logical to determine Zn level in Iranian cirrhotic patients as an important trace element. There are some differences in the etiology, disease progress, prognosis and treatment plan of hepatitis B and C, and it is possible that plasma zinc concentration differs in two groups. The aims of the present study were 1) determination of plasma zinc level in a sample of Iranian cirrhotic patients due to hepatitis B or C, 2) to determine if there is any difference between plasma zinc levels between the 2 groups.

## Materials and Methods

In a cross sectional study, adult cirrhotic inpatients due to hepatitis B or hepatitis C referred to gastrointestinal and liver disease center of Taleghani Hospital, Tehran, Iran were enrolled for the study. This place is one of the important referral centers of liver disease in Iran. Diagnosis of cirrhosis and hepatitis were determined by clinical, laboratory and liver biopsy from the patients. Exclusion criteria were patients who had been co-infected with hepatitis B and C and those who had taken a zinc tablet or any complementary medicine with zinc in its content. Because of the difference in the zinc amount of foods, we preferred to select patients nourished with a similar regimen prepared by the kitchen of the hospital during the study. After informed consent, 5 cc blood samples were kept from the forearm of each patient in fasting state in the morning. Blood samples were centrifuged in 5000 rpm for 5 minutes and plasma was separated. Then plasma samples were kept in the Frazer at the -25 degree centigra  . Because the usual method for assessment of zinc in plasma or serum is the atomic absorption [7], we selected the same method for zinc assessment. Concentrations of 0.1, 0.3, 0.5 and 0.7 ppm of zinc sulfate were prepared as standard samples. Atomic absorptions of them were determined for drawing standard curve. After completion of 60 samples of patients, plasma zinc levels were evaluated by using atomic absorption (Perkin Elmer 1100B).

## Results

60 cirrhotic patients due to hepatitis B or Hepatitis C were included in the study during 7 months (April 2008 to November 2008). Of them 36 were hepatitis B patients and 24 were hepatitis C patients. Demographic data of patients including age distribution, sex and smoking habits were shown in Table 1 Analysis of chi-square showed that there is not any difference between 2 groups regarding to sex, age distribution and smoking habits (P = 0.82, 0.53 and 0.8 respectively).

Mean ± standard deviation (SD) plasma zinc levels determined 0.34±0.22 mg/L and 0.37±0.22 mg/L in hepatitis B and hepatitis C patients respectively. Analysis of t-test showed that there is not any significant difference between 2 groups regarding to plasma zinc level (P = 0.745).

**Table 1:** Demographic of patients.

Parameter		Total Parameter N= 60	HCV Patients (N= 24)	HBV Patients (N=36)	P-value
Age	< 40	12	6	6	0.53
	41-60	40	14	26	
	61>	8	4	4	
Sex	Female	21	8	13	0.82
	Male	39	16	23	
Smoking Habits	No	22	10	12	0.80
	Yes	38	14	24	

## Discussion

Plasma trace elements concentrations are frequently reported to be a good indicator for diagnosis and prognosis of some diseases [8]. Previous studies showed a decrease in zinc level in cirrhotic patients. Pramoolsinsap et al. have stated that serum zinc levels were significantly decreased in patients with chronic active hepatitis, cirrhosis, and hepatocellular carcinoma [9]. Lin et al. announced that the zinc concentration in the serum of Chinese patients with hepatic cirrhosis was significantly less than a control group [10]. It should be noted that zinc level is usually related to the nutritional pattern of each population. It has been shown that zinc deficiency is widespread in people living in developing countries like Iranian populations who consume rice-based diets [11]. The phytate and fiber present in cereal diets can form insoluble complexes with zinc leading to its decreased bioavailability [12].

Since nutritional impairment is common in cirrhotic patients [13], it seems that determination of zinc level in Iranian cirrhotic patients due to hepatitis B and C and comparison with a normal amount of healthy people is necessary as an indicator of nutritional status.

Some demographic data may alter zinc plasma concentration in human. Lopez et al. reported that Serum Zn concentrations were slightly higher in men than in women and also there is some elevated serum Zn levels in smoking men rather than non smokers [14]. In the present study, since there is no significant difference between the 2 groups regarding gender, age distribution and smoking habits, these parameters could not lead to biases in interpretation of zinc level in both of the groups.

The results showed that plasma zinc levels of both groups were below the normal range as mentioned by the similar investigations [9][10]. There are some differences in the normal range of zinc in various populations, but a recent study reported the normal range of 0.89 ± 0.16 mg/L for plasma zinc in healthy volunteers in Tehran [15]. With a comparison result of the present study (0.34±0.22 mg/L and 0.37±0.22 mg/L) with a range of the latter study, it seems that plasma zinc level of the cirrhotic patients are less than half of normal values. As nutritional parameters, this study significantly indicates a zinc level deficiency in Iranian cirrhotic patients due to hepatitis B or C in comparison with healthy volunteers.

The results of the study are more considerable because of the effective role of the zinc supplement in pharmacotherapy of viral hepatitis. Yuasa et al. have shown that zinc may play an important role as a negative regulator of hepatitis C virus (HCV) replication in genome-length HCV RNA-replicating cells. They mentioned that zinc appears to offer a novel approach to the development of future plans for the treatment of intractable chronic hepatitis C [16]. Himoto et al. examined the effects of polaprezinc, a complex of zinc and L-carnosine, on inflammatory activity and fibrosis in the HCV infected patients. They reported that polaprezinc exerts an anti-inflammatory effect on the liver in patients with HCV-related Chronic liver disease by reducing iron overload [17].

Based on the result of the study, administration of zinc may be recommended for Iranian cirrhotic patients due to hepatitis B or C.

In future more studies recommend for the role of zinc administration on clinical, pathological status and pharmacotherapy response of Iranian cirrhotic patients due to hepatitis B or C.
